# Supplementary material for: Enhanced protection face masks do not adversely impact thermophysiological comfort
Source: PLoS One. 2022 Apr 7;17(4):e0265126. doi: 10.1371/journal.pone.0265126 (PMC8989302; doi:10.1371/journal.pone.0265126)
Supplement: Attachment — Submitted filename: Response to Reviewers.pdf [file pone.0265126.s002.pdf]

Dear Reviewers,

Thank you for reviewing our manuscript. Your comments were very helpful and we've improved the manuscript in light of your concerns and suggestions. Please see our responses below response in blue font. Your original comments have been reproduced in black font.

Q: Out of 12 combinations, only one is significant at p<0.05 and only one is not significant (D-2 vs. D-3). What about others.

A: The two p values highlighted in Figure 2 are specifically comparing the effect of surface finish (D-2 vs D-3) and comparing an N95 filter to a regular filter (T-5 vs T-6, respectively). Though not directly displayed in Figure 2, comparing evaporative and thermal resistances of the face masks using ANOVA showed that the addition of the N95 middle layer filter to D-1, D-2, D-3, D-4 and D-5 has a statistically insignificant effect on the thermal resistances to face masks T-1, T-2, T-3, T-4 and T-5, respectively, as well as their evaporative resistances except for face masks T-2 and T-3 (p-values<0.05). This is discussed in the text on pg 14 but would be too messy to display completely within Figure 2.

Q: The introduction section may be condensed by 30-50%.

A: The manuscript has been revised and around 30 lines of text (roughly one page) have been removed from the introduction in order to help focus the manuscript.

Q: Add latest references as the majority of references are more than 5-year-old.

A: The following recent references have now been added to the manuscript:

1. Asadi S, Wexler AS, Cappa CD, Barreda S, Bouvier NM, Ristenpart WD. Aerosol emission and superemission during human speech increase with voice loudness. Scientific Reports. 2019;9:10.

2. Netz RR. Mechanisms of Airborne Infection via Evaporating and Sedimenting Droplets Produced by Speaking. The journal of physical chemistry B. 2020;124(33): 7093-101.

3. Duncan S, Bodurtha P, S N. The protective performance of reusable cloth face masks, disposable procedure masks, KN95 masks and N95 respirators: Filtration and total inward leakage. PLoS ONE. 2021;16(10): e0258191.

4. Lee KP, Yip J, Kan CW, Chiou JC, Yung KF. Reusable Face Masks as Alternative for Disposable Medical Masks: Factors that Affect their Wear-Comfort. Int J Environ Res Public Health. 2020;17(18).

5. Georgi C, Haase-Fielitz A, Meretz D, Gasert L, Butter C. The Impact of Commonly-Worn Face Masks on Physiological Parameters and on Discomfort During Standard Work-Related Physical Effort. Deutsches Arzteblatt International. 2020;117(40): 674-5.

6. Scarano A, Inchingolo F, Lorusso F. Facial Skin Temperature and Discomfort When Wearing Protective Face Masks: Thermal Infrared Imaging Evaluation and Hands Moving the Mask. Int J Environ Res Public Health. 2020;17(13): 9.

7. Clapp PW, Sickbert-Bennett EE, Samet JM, Berntsen J, Zeman KL, Anderson DJ, et al. Evaluation of Cloth Masks and Modified Procedure Masks as Personal Protective Equipment for the Public During the COVID-19 Pandemic. Jama Internal Medicine. 2021.

8. Ke Y, Wang FM. An Exploration of Relationships among Thermal Insulation, Area Factor and Air Gap of Male Chinese Ethnic Costumes. Polymers. 2020;12(6).

Q: Multiple comparison tests revealed few interactions. Authors may try to delete non-significant combinations.

A: Multiple comparisons between the evaporative and thermal resistances of the face masks using ANOVA revealed that the additional N95 middle layer filter had a statistically insignificant effect on the thermal resistances of all the face masks, as well as their evaporative resistances except for face masks T-2 and T-3 (P-values<0.05). The non-significant combinations show the extra N95 filter added can be used in the face mask structure without interfering their thermo-physiological comfort. The lack of statistical significance observed for various mask combinations in this study was very important as it shows how the additional protection offered by the N95 filter did not decrease user comfort. As this is an important finding for all combinations, removing data would not be appropriate.

Q: Any logic of combining 3 different masks T-1 to T-6 and Q-1.

A: As we state on pg7:

"Face masks D 1 to D-6 were two-layer commercially available face masks. To investigate the effect of an additional N95 filter (fabric G, Table 1) on the thermophysiological comfort of the commercially available face masks (D 1 to D 6), face masks T-1 to T-6 were constructed as three layer face masks."

And on pg15:

"Four-layer face mask Q 1 utilized a 50%cotton/50%polyester woven fabric as a face fabric with three underlying knitted 100%cotton layers. This fabric construction has been commercially used as a reusable N94 filter for respirators. Here, the Q-1 fabric system was made into a face mask to see the effect of multiple layering on the evaporative and thermal resistance of the face mask."

So, to summarize, face masks T-1 to T-6 evaluated the addition of an N95 filter, and Q-1 was constructed as a representative N94 filter for respirators.
