# Supplementary material for: Enhanced protection face masks do not adversely impact thermophysiological comfort
Source: PLoS One. 2022 Apr 7;17(4):e0265126. doi: 10.1371/journal.pone.0265126 (PMC8989302; doi:10.1371/journal.pone.0265126)
Supplement: Attachment — Submitted filename: Response to Reviewers.pdf [file pone.0265126.s003.pdf]

Hanna Landenmark
Senior Editor, *PLOS ONE*

Dear Dr. Landenmark,

Thank you for sending us the reviewer comments. They were very helpful and we've improved the manuscript in light of the reviewer concerns and comments. Please see our responses below response in blue font. The original comments have been reproduced in black font.

Q: You have selected the fabric H and Fabric I but not used in the final fabric selection of fabric face mask development. Why?

A: Fabrics H and I were used in the construction of face mask Q-1. This was mentioned in Table 4 but was incorrectly labeled in Table 2, which has been corrected in the updated manuscript.

Q: What is the reason of higher AP of D-3 as compared to D-4 in wet condition as both have same fabric structure?

A: We believe the reviewer is referring to fabrics D-2 and D-3 as they have the same fabric structure unlike D-3 and D-4. The reason for the higher AP of D-2 as compared to D-3 is that D-2 has the Durable Water Repellent finish whereas D-3 does not. As we state on page 13-14:

"The air permeability of fabrics D-2 and D-3 decreased significantly, but was retained in the wet condition. This suggested that the use of knitted fabric structures in face masks could reduce breathing resistance for the wearer in the wet condition. However, fabric D-2 was 75% more air-permeable than fabric D-3 in the dry condition. The considerably lower air-permeability of D-2 in the wet condition was attributed to the durable water repellent finish. Fabric B's inherently greater hydrophobicity in fabric system D-2, compared to fabric C in D-3, resulted in less adhesion of moisture in D-2, allowing for greater air permeation. This demonstrated that water repellent finishing can also improve the thermophysiological comfort of masks."

Q: Please provide the layer-to-layer configuration of the masks and pictures of original developed samples.

A: We have now added Fig 2 which represents the face masks used in the study and their fabric systems. The configuration of layers is now provided in Tables 2 and 4.
